# Anaplastic testicular seminoma presenting as a hydrocele, in a 36-year-old patient with a family history of anaplastic seminoma

**DOI:** 10.15190/d.2022.8

**Published:** 2022-06-30

**Authors:** Orestis Ioannidis, Anastasia Malliora, Panagiotis Christidis, Lydia Loutzidou, Elissavet Anestiadou, Savvas Symeonidis, Nikolaos Ouzounidis, Vassilis Foutsitzis, Ioannis Mantzoros, Stamatios Angelopoulos

**Affiliations:** ^1^4th Academic Department of General Surgery, Aristotle University of Thessaloniki, General Hospital of Thessaloniki “G. Papanikolaou”, Thessaloniki, Greece

**Keywords:** Testicular cancer, seminoma, anaplastic cancer, hydrocele, family history of seminoma.

## Abstract

Testicular cancer is the solid tumor with the greatest incidence in men between 15 and 44 years old. Its main histological type is germ cell tumor, that is divided into seminomatous and non-seminomatous tumors. Seminoma, consisting 55% of testicular cancer, manifests in the fourth decade of men’s life and a rare type of it is the anaplastic seminoma (5% to 15% of testicular seminoma). Diagnosis is based on clinical examination, testicular ultrasound, magnetic resonance imaging, tumor markers measurement and pathological examination, while treatment of choice is radical orchiectomy, with or without retroperitoneal lymphadenectomy, potentially followed by radiotherapy or chemotherapy. We present the case of a 36-year-old male patient, whose father suffered from anaplastic seminoma and visited the hospital due to a painless hydrocele. The testicle was swollen and hard on palpation, while cytological examination of the drained fluid detected neoplasm, potentially seminoma. Tumor markers measurement, as well as abdominal and pelvic computerized tomography scans, were evaluated and the patient was staged as IIA, according to the American Joint Commission on Cancer. Radical orchiectomy with high ligation of the seminal vesicle was performed and the pathological examination showed anaplastic testicular seminoma. Postoperatively, four cycles of chemotherapy with bleomycin, etoposide and platinum (BEP) were performed and no signs of recurrence were present after 1 year. In conclusion, anaplastic seminoma has a good prognosis and is suggested to be treated with radical orchiectomy, with or without retroperitoneal lymphadenectomy, potentially followed by radiotherapy or chemotherapy.

## INTRODUCTION

In Western countries, testicular cancer accounts for 1% of male malignancies and 5% of urological cancers. Its main histological type is germ cell tumor (GCT), that is divided into seminomatous and non-seminomatous tumors. Seminoma, consisting of 55% of testicular cancer is most commonly manifested in the fourth decade of men’s life as a palpable mass and a rare type of it, documented by a few publications in the literature, is anaplastic seminoma (5% to 15% of testicular seminoma)^1-3^.We present the case of a 36-year-old male patient with a family history of anaplastic seminoma who suffered from anaplastic seminoma and was successfully treated with radical orchiectomy followed by chemotherapy. Written informed consent was obtained from the patient, and the institutional review board of our hospital approved this report. The following case report is presented in accordance with Surgical CAse REport (SCARE) guidelines^4^.

## CASE REPORT

A 36-year-old patient, with a family history of anaplastic seminoma from his father, came to the hospital with a painless hydrocele that presented at least two months before. Palpation revealed swelling and hardness of the testicle. Drainage was performed. The cytological examination of the fluid detected a neoplasm and a seminoma was suspected.

During the preoperative examination, blood and imaging tests were performed. Alpha-fetoprotein (AFP) (normal values: 0 and 8 ng/mL) and lactate dehydrogenase (LDH) values (normal values: 140 to 280 U/L) were within normal limits, whereas beta-human chorionic gonadotropin (β-hCG) was elevated 600IU/L (normal values: 0.02-0.8IU/L). Chest radiography was normal, whereas pelvic and abdominal computerized tomography (CT) scans showed a small number of swollen inguinal and paraaortic lymph nodes below the level of the renal arteries and the patient was staged as II according to the American Joint Commission on Cancer (AJCC)^5^.

**Figure 1:**
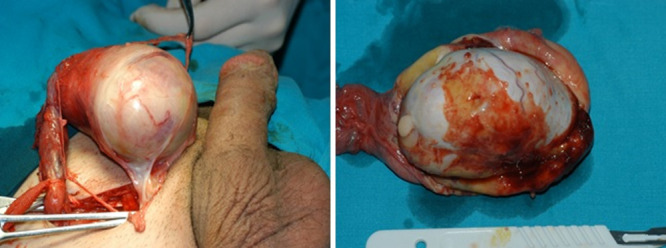
Anaplastic seminoma of the testicle (intraoperative image)

No prior biopsy of the testicular mass was performed due to the risk of tumor seeding via lymphatic drainage. The patient underwent radical orchiectomy in classic fashion with high ligation of the seminal vesicle (Figure 1). Pathological examination of the mass showed nuclear pleomorphism, high mitotic rates and focal areas of necrosis. Thus, it was categorized as an anaplastic testicular seminoma.

 The patient was discharged in the 2^nd^ postoperative day. He underwent four cycles of chemotherapy with bleomycin, etoposide and platinum (BEP). The laboratory and imaging re-examination at 3, 6, 9 and 12 months were within the normal limits, with no sign of recurrence.

## DISCUSSION

In Western countries, although testicular cancer only accounts for 1% of male malignancies and 5% of urological cancers, it is the solid tumor with the greatest incidence in men between 15 and 44 years old. Its main histological type (95%) is germ cell tumor (GCT) that is divided into seminomatous and non-seminomatous tumors. A rare type of seminoma, with a few publications in the literature, is anaplastic seminoma (5% to 15% of testicular seminoma), which is separated from classical seminoma based on the greater mitotic activity, the cellular irregularity, the absence of fibrovascular septae, the presence of a few lymphocytes, the focal necrosis and the pleomorphic cells with non-clear cytoplasm^1-3^.

Seminoma is diagnosed on men with average age of 35-39 years old, as in our case and it mainly presents with a painless, palpable mass and rarely with swelling of the testicles or mild discomfort^2^.In general, risk factors for testicular cancer, including seminoma, includes personal history of testicular GCT, cryptorchidism that remains untreated, family history and environmental factors, such as organochlorines, polyvinyl chlorides, polychlorinated biphenyls, cannabis and smoking^1,2^. Regarding the family factor, men who have a father that has suffered from testicular GCT, as our patient, are 2 to 4 times more likely to develop this type of cancer themselves than the general population, whereas those who have an affected brother are 5 to 19 times more likely to develop testicular GCT^6^. c-KIT ligand allele variation is considered to be the most important genetic risk factor for testicular GCT^7^.As for cryptorchidism, the incidence of testicular cancer in patients with untreated cryptorchidism is 8 times higher than the general population^2^.

Diagnosis is based on clinical examination, testicular ultrasound, MRI, tumor markers measurement (AFP, β-hCG, LDH) and pathological examination^7^.In the case of suspected mass in the testicle, scrotum ultrasonography is the first imaging method that is applied^2^. It can accurately distinguish if the lesion is inside or outside the testicle and can also distinguish benign cystic lesions from other more suspicious solid masses^2^. Seminoma occurs as a hypoechoic, homogeneous mass of various sizes and with increased vascularity in Doppler ultrasonography^2^. On MRI, seminoma occurs as a low-intensity homogeneous mass in T2-weighted images, while in the case of bleeding or necrosis, areas of heterogeneity are observed^2^. CT scans are used to detect possible swollen lymph nodes and especially interaortocaval nodes inferior to the renal hilar vessels in case of right-sided tumor and paraaortic and preaortic lymph nodes in case of left-sided tumors^2^. As to tumor markers, in seminoma AFP values are normal, whereas β-hCG and LDH can be increased^2^. In our patient only β-hCG was elevated.

Nowadays, testicular cancer is diagnosed at an early stage, resulting in high survival rates^1^. In particular, the 3-, 5- and 10-year survival rates for seminoma are 95%, 86%, and 71%, respectively^2^. Anaplastic seminoma is thought to have a prognosis comparable to the classical seminoma, with a 5-year survival rate that ranges from 43% to 95%^8^ or according to others up to 80%^9^. Treatment of choice for testicular GCT, including seminoma, is radical orchiectomy, with or without retroperitoneal lymphadenectomy, that could be followed by radiotherapy or chemotherapy^7^,depending on stage, histology and risk classification^3^. Radiotherapy is performed in stage IIA seminoma of small size and in selective cases of stage IIB, while chemotherapy is chosen for IIB, IIC and III stages and it includes etoposide and cisplatin (EP) or bleomycin, etoposide, and cisplatin (BEP)^2^. Four cycles of chemotherapy with BEP were chosen to be performed on our patient. Anaplastic seminoma treatment is believed to be the same as the one performed for seminoma^1^.Today, new treatments, such as immunotherapy, are being considered^7^.

## CONCLUSION

 In conclusion, seminoma is the most common type of testicular cancer that can be rarely manifested as anaplastic. Despite the limited information about this histological type, anaplastic seminoma is considered to have good prognosis. Family history is a risk factor. Anaplastic seminoma is suggested to be treated as the classic seminoma with radical orchiectomy, with or without retroperitoneal lymphadenectomy, potentially followed by radiotherapy or chemotherapy.
